# How Does Fluid Flow Influence Drug Release from Drug Filled Implants?

**DOI:** 10.1007/s11095-021-03127-4

**Published:** 2022-01-07

**Authors:** David King, Christopher McCormick, Sean McGinty

**Affiliations:** 1 Division of Biomedical Engineering, University of Glasgow, Glasgow, UK; 2 Department of Biomedical Engineering, University of Strathclyde, Glasgow, UK; 3 Glasgow Computational Engineering Centre, University of Glasgow, Glasgow, UK

**Keywords:** fluid dynamics, drug filled implants, drug release, mathematical modelling, porous implants

## Abstract

Drug-filled implants (DFIs) have emerged as an innovative approach to control the delivery of drugs. These devices contain the drug within the structure of the implant itself and avoid the need to include additional drug carrier materials such as a polymers, which are often associated with inflammation and delayed healing/tissue regeneration at the implant site. One common feature of *in vitro* experiments to generate drug release profiles is stirring or agitation of the release medium. However, the influence of the resulting fluid flow on the rate of drug release from DFIs has yet to be quantified. In this paper we consider two DFIs, which although similar in shape and size, employ different strategies to control the release of drug: a porous pin with pores on the order of *μ*m and a pin drilled with orifices of the order of mm. We develop a multiphysics mathematical model of drug release from these DFIs, subject to fluid flow induced through stirring and show that fluid flow greatly influences the drug release profile for the orifice pin, but that the porous pin drug release profile is relatively insensitive to flow. We demonstrate that drug release from the porous pin may adequately be described through a simplified radial 1D dissolution-diffusion model, while a 3D dissolution-advection-diffusion model is required to describe drug release from the orifice pin. A sensitivity analysis reveals that that the balance of reaction-advection-diffusion in terms of key nondimensional numbers governs the overall drug release. Our findings potentially have important implications in terms of devising the most relevant experimental protocol for quantifying drug release from DFIs.

**Supplementary Information:**

The online version contains supplementary material available at 10.1007/s11095-021-03127-4.

## Introduction

Drug-filled implants (DFIs) have emerged as a novel technology that enable localised drug delivery without the need for a polymer drug carrier. This is potentially advantageous since polymer-coated implants have been associated with adverse effects such as lack of biocompatibility, inflammation, or delayed healing/tissue regeneration at the implant site (1–3). Instead, DFIs incorporate the drug within the structure of the implant itself. The drug is typically contained within one or more reservoirs, with the surrounding material porosity (on varying length scales) being a key design parameter in terms of controlling the rate of drug release. DFIs are being investigated in several application areas, with prototype devices having already been designed for cardiovascular and orthopaedic applications, for example, drug-filled coronary stents (4) and drug-filled fixation pins (5, 6), respectively.

Early-stage testing of drug-delivery implants usually involves a series of *in vitro* experiments, primarily to explore the effect of different design configurations and to test the repeatability of the drug release profile. Only the most promising designs are taken forward to *in vivo* studies and eventually clinical trials, primarily because of the prohibitive costs and associated ethical issues. Therefore, it is of paramount importance that the *in vitro* testing is performed under the most suitable experimental conditions. Unfortunately, experimental protocols for such testing vary widely in the literature, meaning that it is often difficult to make comparisons and, more importantly, extrapolate the *in vitro* drug release profile to the more complex *in vivo* setting.

The features of *in vitro* experimental protocols that typically differ can be roughly separated into five areas:

### (i) Selection of Release Media

The release media selected can strongly influence the drug release kinetics observed *in vitro*. Phosphate Buffered Saline (PBS) maintained at 37 °C is often used to mimic physiological conditions. The release of protein-binding drugs can be modified by the presence of proteins within release media. For characterisation of hydrophobic drug release, the media will often include agents to enhance solubility in order to better capture *in vivo* release, with the inclusion of ethanol (7) and Tween (8) having been reported. In common with the methods used for dissolution testing of more conventional pharmaceutical dosage forms, accelerated release studies can be performed through selection of release media with maximal drug solubility. The extent to which the release profiles generated from such modifications allows accurate predictions of *in vivo* drug release remains unclear.

### (Ii) Release Volume

The volume of the release media greatly impacts upon *in vitro* drug release characteristics. It is often recommended that such experiments are performed under infinite sink conditions (9), thereby necessitating the use of relatively large media volumes. Smaller volumes may be selected, which can make analytical measurement of the drug concentrations less challenging, but given that this will give rise to temporal changes in the media drug concentration, this requires careful consideration when characterising systems where diffusion is a dominant release mechanism.

### (Iii) Sampling Method

Continuous *in situ* monitoring of *in vitro* drug release from medical implants is rarely reported, with periodic sampling more typically used to allow drug measurements to be performed on standard analytical instruments. Periodic sampling in this way allows the media to be refreshed at each time point, with complete removal of the media and replacement often selected in order to maintain infinite sink conditions as closely as possible. However, partial replacement of media has also been reported. It is clear that the evolution of drug concentration within the release media will be impacted by the frequency of sampling and the media replacement strategy, with this in turn having potentially important effects on the drug release kinetics, particularly where diffusion is the dominant transport mechanism (10, 11).

### (Iv) Geometry Effects

The release media volume selected governs the choice of incubation vessel used, with the geometry of this in turn potentially impacting upon the drug release kinetics. Cylindrical glass vials are widely used, particularly for examination of drug release from small medical implants such as vascular stents (12). Flasks of various geometries are also used, permitting the introduction of stirred regimes. The geometry of the implant material and its placement within the incubation vessels will also impact upon drug release characteristics. The drug-coated surface should be exposed uniformly to the release media, although this is not always achieved in systems where the samples are placed at the bottom of the vessel.

### (v) Stirring/Agitation Conditions

The release media can be stirred or unstirred. For conventional dosage forms, standardised dissolution testing apparatus is used in accordance with United States Pharmacopeia (USP) standards (13). The standards governing the evaluation of drug release kinetics from implantable devices rely on similar testing apparatus and protocols, although are less well defined (14). For vascular implants that are exposed to blood flow, perfusion systems are often used to characterise drug release (15) although more simplified agitation systems have also been employed (7). Gentle agitation can be provided to mimic movement of extracellular fluid around other implantables, such as orthopaedic or subcutaneous devices. The nature of the *in vitro* flow regime achieved will be governed by all of the factors outline above to some extent, although the precise impact of this upon the drug release characteristics remains unclear.

In this article, we shall primarily be concerned with *(v)*-stirring/agitation conditions. Specifically, through the development of a multiphysics mathematical model applied to two DFIs with quite different material properties, we seek to understand the influence of fluid flow on drug release from these DFIs.

## Test Cases

**Fig. 1:**
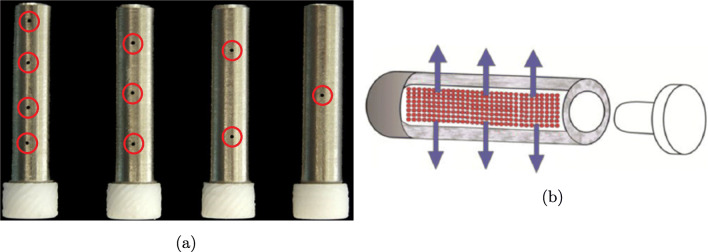
(a) The *orifice pins* showing (left to right) 8, 6, 4 and 2 drilled holes, highlighted by the red circles, through which the drug is released (6). (b) Illustration of the *porous pin* with blue arrows showing drug transport through the porous wall (5).

In this study, we consider two different prototype drug-filled orthopaedic-fixation pins (5, 6) as shown in Fig. 1. Each pin is made of stainless steel and comprises a cylindrical geometry with a cylindrical hollowed-out portion acting as a reservoir filled with dry drug. The geometrical parameters of the pins are similar and are summarised in Table 1. The key difference between the pins is related to the drug release route: the first (the *orifice pins*, Fig. 1a) contains a small number (2-8) of large orifices on the surface, while the second (the *porous pin*, Fig. 1b) encompasses a homogeneous porous wall.

**Table 1:** Geometrical parameters of the pins considered

Geometrical feature	Orifice Pins (6)	Porous Pin (5)
Radius of drug filled core	1.4 mm	1*.*575 mm
Wall thickness	1.6 mm	1*.*6 mm
Pin length	25 mm	25*.*4 mm
Outer diameter	6 mm	6*.*35 mm
Wall porosity	N/A	0*.*17
Orifice diameter	0.5 mm	N/A

## Mathematical Model Formulation

### The Orifice Pins

#### Geometrical Model

**Fig. 2:**
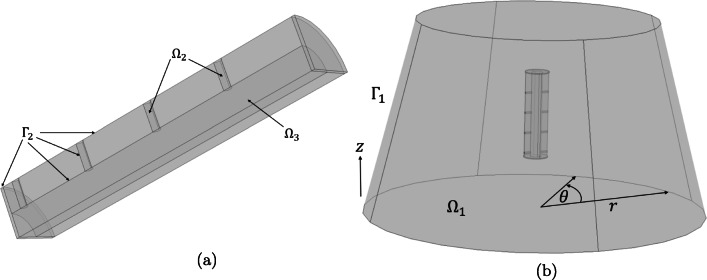
(a) A close-up of a segment of the 8-orifice pin, clearly showing the drug core ${\Omega}_3$ and the collection of orifices ${\Omega}_2$, which extend through the wall of the pin. ${\Gamma}_2$ represents the entire surface area of the pin, including the cylindrical walls of the orifices in each pin configuration. (b) Geometrical configuration of the 8-hole pin within a section of a conical flask. The release medium fluid is represented by ${\Omega}_1$. The set of boundaries between the release medium and the flask are collectively represented by ${\Gamma}_1$. The default Cartesian (*x*,*y*,*z*) co-ordinate system within COMSOL Multiphysics^®;^, version 5.3a, was employed, with the origin located at the centre of the flask base and the *z*-axis pointing vertically upwards. On the boundary ${\Gamma}_1$, a moving wall boundary condition (2) is imposed to simulate stirring. For ease of implementation, a cylindrical co-ordinate system was employed for this boundary condition: *r* denotes the radius from the central $z$-axis to the outer wall and *θ* is the angle which ${\Omega}_1$ makes with respect to its original position as it rotates.

Although four versions of the orifice pin were created by Gimeno et al. (6), we consider here only the 2-orifice and 8-orifice pins, representing the extreme cases. The orifice pins (Fig. 2) comprise a central drug-filled hollow core (${\Omega}_3$) connected to orifices (${\Omega}_2$) drilled through the cylindrical wall of the pins. Following correspondence with the authors, it was revealed that the release medium and orifice pins were contained within 250 mL conical flasks, whose dimensions are available from the manufacturer (16). Although the release medium vessel is a conical flask, only the volume occupied by the release medium is required for the computational modelling and so the neck of the conical flask is not included in the computational geometry. Moreover, the authors confirmed that the pins were suspended in the release media. The geometry of each pin and release container was constructed in COMSOL Multiphysics®, version 5.3a, where the default Cartesian (*x, y, z*) co-ordinate system was employed. In Fig. 2 we display the geometries associated with the 8-orifice pin, highlighting the notation used to define each domain and boundary.

#### Fluid dynamics

While the release medium used in (6) is known to be Simulated Body Fluid (SBF), we have been unable to identify the fluid properties (e.g. density and kinematic viscosity) of SBF in the literature. For the purposes of this study, we assume that SBF has similar properties to those of water and, therefore, describe the fluid dynamics in the container and within the pin using the incompressible Navier-Stokes equations (1) supplemented with appropriate boundary and initial conditions (2-4):

1 $$ {\displaystyle \begin{array}{lcrr}\frac{\partial \boldsymbol{u}}{\partial t}& =& \nu {\nabla}^2\boldsymbol{u}-\left(\boldsymbol{u}\cdot \nabla \right)\boldsymbol{u}-\frac{1}{\rho}\nabla p,\kern1em \nabla \cdot \boldsymbol{u}=0,& \\ {}& & \mathrm{in}\kern2.77695pt {\Omega}_1,{\Omega}_2\kern2.77695pt \mathrm{and}\kern2.77695pt {\Omega}_3,\kern1em t>0,\end{array}} $$

2 $$ \boldsymbol{u}=\left(-r\sin \left(\theta \right),r\cos \left(\theta \right),0\right)\omega, \kern1em \mathrm{on}\kern2.77695pt {\Gamma}_1,\kern1em t>0, $$

3 $$ \boldsymbol{u}=\boldsymbol{0},\kern1em \mathrm{on}\kern2.77695pt {\Gamma}_2,\kern1em t>0, $$

4 $$ \boldsymbol{u}=\boldsymbol{0},\kern1em \mathrm{in}\kern2.77695pt {\Omega}_1,{\Omega}_2\kern2.77695pt \mathrm{and}\kern2.77695pt {\Omega}_3,\kern1em t=0, $$

where ***u*** denotes the fluid velocity field, assumed to be zero initially (4), $\rho$ is the fluid density, $p$ is the pressure and $\nu$ is the kinematic viscosity of the fluid. On the boundary Γ_1_, a moving wall boundary condition (2) is imposed. For ease of implementation, a cylindrical co-ordinate system was employed for this boundary condition, in which $\omega$ is the magnitude of the angular velocity of the rotating wall in rad/s. This condition ensures that the fluid at this boundary takes the wall’s angular velocity: this is the driving force behind the fluid flow. A no-slip/no-penetration boundary condition (3) is applied to the collection of boundaries that comprise the body of the pin, denoted by Γ_2_. We note that this model inherently assumes that the submerged pin is filled with fluid at *t* = 0. In reality, the SBF will take some finite time to infiltrate the pin and will depend on many factors including the diameter of the orifices and the structure of the (porous) dry drug core. In cases where the timescale associated with drug dissolution is significant, this model may represent an under-estimation of the drug release time.

#### Drug transport

Drug transport within the release medium may in principle be governed by advection as a result of fluid flow as well as diffusion as a result of random molecular motion. Additionally, when the drug is loaded in a dry solid form, as is the case in Gimeno et al. (6), the rate of dissolution of drug may be a key driver of drug release. Indeed, (6) considered in their study two commercially available drug products with differing solubilities (Cefazolin Sodium and Linezolid, with solubilities in water of 50 mg/mL and 3 mg/mL, respectively), noting significant differences between the drug release profiles. Thus, to enable the possibility of each of these mechanisms being equally important, we propose a dissolution-diffusion-advection model to describe drug transport within the hollow interior of the pin:

5 $$ \frac{\partial b}{\partial t}=-\beta {b}^{2/3}\left(S-c\right),\kern1em \mathrm{in}\kern2.77695pt {\Omega}_3,\kern1em t>0, $$

6 $$ \frac{\partial c}{\partial t}={D}_f{\nabla}^2c-\boldsymbol{u}\cdot \nabla c+\beta {b}^{2/3}\left(S-c\right),\kern1em \mathrm{in}\kern2.77695pt {\Omega}_3,\kern1em t>0, $$

where $b\left(x,y,z,t\right)$ and $c\left(x,y,z,t\right)$ are the concentrations of undissolved and dissolved drug, respectively, $S$ is the solubility of the drug in the release medium, $\beta$ is the drug dissolution rate, and ${D}_f$ is the isotropic free-diffusion coefficient of the drug in the release medium. This nonlinear dissolution model was originally proposed by (17) and represents a modification of the classical Noyes-Whitney model (18) allowing for the possibility of the dissolution rate being a function of the surface area of spherical dissolving drug particles. The model (5-6) assumes that the orifice pins are rapidly infiltrated by the release medium fluid and that the drug is fully wetted. This enables the dissolution process to begin throughout the drug core immediately. In reality, dissolution is a complex process consisting of several steps. There are more complex models in the literature (19) which account for each individual step, highlighting the importance of the initial wetting stage. The equation proposed to model mass transport in the bulk fluid domain and in the orifices is:

7 $$ \frac{\partial c}{\partial t}={D}_f{\nabla}^2c-\boldsymbol{u}\cdot \nabla c,\kern1em \mathrm{in}\kern2.77695pt {\Omega}_1\mathrm{and}{\Omega}_2,\kern1em t>0. $$

We impose the following boundary and initial conditions

8 $$ \hat{\boldsymbol{n}}\cdot \left(-{D}_f\nabla c+c\boldsymbol{u}\right)=0,\kern1em \mathrm{on}\kern2.77695pt {\Gamma}_1,\kern1em t>0, $$

$$ -\hat{\boldsymbol{n}}\cdot {D}_f\nabla c=0,\kern1em \mathrm{on}\kern2.77695pt {\Gamma}_2,\kern1em t>0, $$

9 $$ b={b}_0,\kern1em \mathrm{in}\kern2.77695pt {\Omega}_3,\kern1em t=0, $$

10 $$ c=0,\kern1em \mathrm{in}\kern2.77695pt {\Omega}_1,{\Omega}_2\kern2.77695pt \mathrm{and}\kern2.77695pt {\Omega}_3,\kern1em t=0, $$

where $\hat{\boldsymbol{n}}$ is the outward facing unit normal to applicable boundary surfaces and ${b}_0$ is the initial drug loading concentration.

### The Porous Pin

#### Geometrical Model

**Fig. 3:**
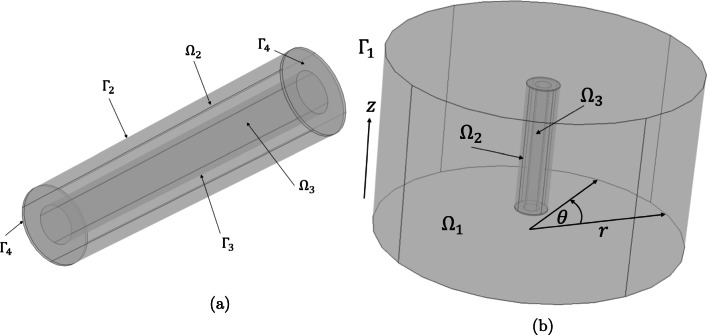
(a) The 3D porous pin highlighting ${\Omega}_2$ (the porous wall) and ${\Omega}_3$ (the inner drug core). The outer boundary of the porous wall of the pin in contact with the release medium is denoted ${\Gamma}_2$ and the interface between the inner drug core and the porous wall is represented by ${\Gamma}_3$. The collection of boundaries that form the top and bottom caps of the pin are designated by ${\Gamma}_4$. (b) 3D geometry of the porous pin experimental set-up, where the domains ${\Omega}_1$, ${\Omega}_2$ and ${\Omega}_3$ represent the release medium, the porous wall of the pin and the inner drug core of the pin, respectively. ${\Gamma}_1$ is the outer boundary of the release medium, *θ* is the angle that ${\Omega}_1$ makes with respect to its original position as it rotates and *r* is the radius from the central *z*-axis to the outer wall.

The porous pin is comprised of two distinct regions: the inner drug core (Ω_3_) and the porous wall (Ω_2_) as shown in Fig. 3a. In the absence of any information to suggest otherwise, the container for the release medium is assumed to be a beaker of radius 30 mm (20). Since the volume of release medium is known to be 100 ml (5), the depth of the release medium may be easily calculated to provide as accurate a representation as possible for the model. The porous pin geometry (Fig. 3a) is then suspended in the centre of the beaker geometry as shown in Fig. 3b. This configuration was motivated through correspondence with the authors of the original work detailing the prototype pins (5). The geometry of the porous pin and release container were constructed in COMSOL Multiphysics®, version 5.3a, where the default Cartesian (*x, y, z*) co-ordinate system was employed. The notation used to define each domain and boundary is show in Fig. 3.

#### Fluid Dynamics

As with the orifice pins experiments, the release medium is SBF (5) with fluid dynamic properties assumed similar to water. The time-dependent incompressible Navier-Stokes equations (11,13) are used to model fluid flow in the bulk release medium and the inner drug core of the pin. However, these equations are not appropriate for fluid modelling in the porous wall of the pin (${\Omega}_2$), where we instead impose the time-dependent incompressible Brinkman equations (12,13). The Brinkman equations, preferred to Darcy’s law because of their more accurate approximation of bulk fluid-porous domain transitions (21,p. 16), introduce two additional parameters: $\kappa$, the permeability of the porous wall of porosity $\phi$. respectively. Furthermore, ν is the kinematic viscosity of the fluid (the ratio of dynamic viscosity to density). The fluid dynamics model is then given by:

11 $$ \frac{\partial \boldsymbol{u}}{\partial t}=\nu {\nabla}^2\boldsymbol{u}-\left(\boldsymbol{u}\cdot \nabla \right)\boldsymbol{u}-\frac{1}{\rho}\nabla p,\kern5pt \mathrm{in}\kern2.77695pt {\Omega}_1\mathrm{and}\kern2.77695pt {\Omega}_3,\kern1em t>0, $$

12 $$ \frac{1}{\phi}\frac{\partial \boldsymbol{u}}{\partial t}=\frac{\nu }{\phi }{\nabla}^2\boldsymbol{u}-\frac{1}{\phi^2}\left(\boldsymbol{u}\cdot \nabla \right)\boldsymbol{u}\kern5pt -\frac{1}{\rho}\nabla p-\frac{\nu }{\kappa}\boldsymbol{u},\mathrm{in}\kern2pt {\Omega}_2,\kern6pt t>0, $$

13 $$ \nabla \cdot \boldsymbol{u}=0,\kern1em \mathrm{in}\kern2.77695pt {\Omega}_1,{\Omega}_2\kern2.77695pt \mathrm{and}\kern2.77695pt {\Omega}_3,\kern1em t>0. $$

These equations are supplemented with appropriate boundary and initial conditions (14-17):

14 $$ \boldsymbol{u}=\left(-r\sin \left(\theta \right),r\cos \left(\theta \right),0\right)\omega, \kern1em \mathrm{on}\kern2.77695pt {\Gamma}_1,\kern1em t>0, $$

15 $$ {\boldsymbol{u}}_{ns}={\boldsymbol{u}}_{br},\kern1em {p}_{ns}={p}_{br},\kern1em \mathrm{on}\kern2.77695pt {\Gamma}_2\kern2.77695pt \mathrm{and}\kern2.77695pt {\Gamma}_3,\kern1em t>0, $$

16 $$ \boldsymbol{u}=\boldsymbol{0},\kern1em \mathrm{on}\kern2.77695pt {\Gamma}_4,\kern1em t>0, $$

17 $$ \boldsymbol{u}=\boldsymbol{0},\kern1em \mathrm{in}\kern2.77695pt {\Omega}_1,{\Omega}_2\kern2.77695pt \mathrm{and}\kern2.77695pt {\Omega}_3,\kern1em t=0. $$

The definitions of $\boldsymbol{u}$, $\rho$, $p$, ν and $\omega$ are the same as with the orifice pins case. As before, a moving wall boundary condition (14) is used. Also, a no-slip/no-penetration boundary condition (16) is applied along with the assumption that the release medium is at rest initially (17). At the boundaries $\boldsymbol{u}$, $\rho$, $p$, ν and $\omega$we impose continuity of velocity and pressure (15), where the subscripts $ns$ and $br$ indicate the velocity field and pressure associated with Navier-Stokes ($ns$) and Brinkman ($br$), respectively.

#### Drug Transport

The fluid dynamic equations (11-17) are coupled with a set of reaction-diffusion-advection equations (18-21). The drug concentration in the inner drug core (${\Omega}_3$), the porous wall of the pin (${\Omega}_2$) and the bulk release medium (${\Omega}_1$) are denoted ${c}_d\left(x,y,z,t\right)$, ${c}_p\left(x,y,z,t\right)$ and ${c}_m\left(x,y,z,t\right)$, respectively. The proposed model is:

18 $$ \frac{\partial b}{\partial t}=-\beta {b}^{2/3}\left(S-c\right),\kern1em \mathrm{in}\kern2.77695pt {\Omega}_3,\kern1em t>0, $$

19 $$ {\displaystyle \begin{array}{lcrr}\frac{\partial {c}_d}{\partial t}& =& {D}_f{\nabla}^2{c}_d-\boldsymbol{u}\cdot \nabla {c}_d+\beta {b}^{2/3}\left(S-c\right),& \\ {}& & \mathrm{in}\kern2.77695pt {\Omega}_3,\kern1em t>0,\end{array}} $$

20 $$ \phi \frac{\partial {c}_p}{\partial t}={D}_p{\nabla}^2{c}_p-\boldsymbol{u}\cdot \nabla {c}_p,\kern1em \mathrm{in}\kern2.77695pt {\Omega}_2,\kern1em t>0, $$

21 $$ \frac{\partial {c}_m}{\partial t}={D}_f{\nabla}^2{c}_m-\boldsymbol{u}\cdot \nabla {c}_m,\kern1em \mathrm{in}\kern2.77695pt {\Omega}_1,\kern1em t>0, $$

where ${D}_p$ is the effective diffusion coefficient within the porous wall, calculated via ${D}_p={\phi}_e{D}_f/\tau$(22). The parameters *ϕ*_*e*_ and *τ* are the effective porosity of the porous wall and the tortuosity, respectively. These governing equations are supplemented with the boundary and initial conditions:

22 $$ {c}_d={c}_p,\kern1em \hat{\boldsymbol{n}}\cdot \left(-{D}_f\nabla {c}_d+{c}_d\boldsymbol{u}\right)=\hat{\boldsymbol{n}}\cdot \left(-{D}_p\nabla {c}_p+{c}_p\boldsymbol{u}\right),\kern1em \mathrm{on}\kern2.77695pt {\Gamma}_3,\kern1em t>0, $$

23 $$ {c}_p={c}_m,\kern1em \hat{\boldsymbol{n}}\cdot \left(-{D}_p\nabla {c}_p+{c}_p\boldsymbol{u}\right)=\hat{\boldsymbol{n}}\cdot \left(-{D}_f\nabla {c}_m+{c}_m\boldsymbol{u}\right),\kern1em \mathrm{on}\kern2.77695pt {\Gamma}_2,\kern1em t>0, $$

24 $$ \hat{\boldsymbol{n}}\cdot \left(-{D}_f\nabla {c}_m+{c}_m\boldsymbol{u}\right)=0,\kern1em \mathrm{on}\kern2.77695pt {\Gamma}_1,\kern1em t>0, $$

25 $$ {\displaystyle \begin{array}{lcrr}\hat{\boldsymbol{n}}\cdot {D}_f\nabla {c}_d& =& \hat{\boldsymbol{n}}\cdot {D}_p\nabla {c}_p=\hat{\boldsymbol{n}}\cdot {D}_f\nabla {c}_m=0,\kern1em & \\ {}& & \mathrm{on}\kern2.77695pt {\Gamma}_4,\kern1em t>0,\end{array}} $$

26 $$ b={b}_0,\kern1em \mathrm{in}\kern2.77695pt {\Omega}_3,\kern1em t=0, $$

27 $$ {\displaystyle \begin{array}{lcrr}{c}_d& =& 0,\kern1em \mathrm{in}\kern2.77695pt {\Omega}_3,\kern1em {c}_p=0,\kern1em \mathrm{in}\kern2.77695pt {\Omega}_2,\kern1em & \\ {}{c}_m& =& 0,\kern1em \mathrm{in}\kern2.77695pt {\Omega}_1,\kern1em t=0.\end{array}} $$

Continuity of concentration and flux conditions (22–23) are applied to boundaries Γ_2_ and Γ_3_. On Γ_1_ and Γ_4_, zero-flux boundary condition (24–25) are applied to prevent drug from leaving the system, with the latter neglecting the advective component due to the zero-flux/zero-penetration conditions imposed on the fluid (16). Initially, there exists only undissolved drug (26) and no dissolved drug (27).

### Summary of Investigated Scenarios

Since the focus is on establishing the influence of flow on drug release, we initially neglect dissolution and solve the corresponding 3D advection-diffusion models with all of the drug assumed to be in the dissolved phase initially at concentration ${b}_0$. This approximation is valid when the drug is either initially in a dissolved form, or is rapidly dissolved (e.g. high dissolution rate and/or solubility). We also consider the case of steady flow versus time-dependent flow. Once the effect of flow is established, we then reintroduce dissolution to the models. Given the geometry of the porous pin, we proceed to explore whether or not it is possible to exploit symmetry to simplify the model. Finally, when we have established the feasible simplifications to the model for each pin, we conduct a sensitivity analysis to explore the effect on drug release of varying the key model parameters. The key measure we use to compare the results is the drug release profile, defined as the mass of drug that has accumulated in the release medium (or released from the pins) at any time, *t*, normalised with respect to the initial drug loading.

### Numerical Solution

The orifice and porous pin model equations (1-27) were nondimensionalised prior to solving numerically and applied to the geometries shown in Figs. 2 and 3, respectively. Additionally, a 2-orifice pin geometry was also considered, with the orifices located as shown in Fig. 1a. All spatial variables were scaled with the radius of the drug core, ${L}_d$. The remaining scalings employed were:

$$ {c}^{\prime }=\frac{c}{S},\kern1em {b}^{\prime }=\frac{b}{b_0},\kern1em {t}^{\prime }=\frac{D_f}{L_d^2}t,\kern1em {\mathbf{u}}^{\prime }=\frac{L_d}{D_f}\mathbf{u},\kern1em {p}^{\prime }=\frac{L_d^2}{\rho {D}_f^2}p. $$

The models were solved using the commercial finite element method (FEM) software, COMSOL Multiphysics®, version 5.3a. There exist several numerical methods within the software, with the default methods for a given combination of physics selected by the software automatically. The particular methods considered for each study are described in the Results and Discussion Section. However, in all numerical studies, time-advancement was handled by the backward differentiation formula (BDF), with free time stepping. To aid in conservation of mass when the drug-transport equations had an advective component, the equations of each study were solved in conservative form. Preliminary modelling suggested that mass conservation could be influenced by the specific values of the parameters. Therefore, suitably dense meshes were constructed for use across all parameters considered. The meshes used in all studies were considered suitable if the error in mass conservation was less than 1%. For the models involving the Navier-Stokes equations, since the pressure is not defined anywhere in the system, a pressure point constraint was imposed by setting the pressure to zero at an arbitrary point in the pin domains to allow the numerical scheme to find a solution.

### Parameter Values

A summary of the baseline parameter values used in this study is provided in Table 2. Several of the parameters have been taken or inferred from (5) and (6). The initial drug concentration and solubility values are based upon the antibiotics used in these studies: Cefazolin for the Orifice pins and Linezolid for the Porous Pin. The drug loading in the case of the orifice pins is explicitly stated (100 mg (6)), therefore, the initial drug concentration can be calculated. However, in the case of the porous pin, the authors state that the porous pins were loaded with 95 *−* 120 mg of the drug for the release experiments. For simplicity, we assume the drug loading is the same as in the case of the orifice pins (100 mg). We now discuss the remaining parameters.

**Table 2:** Baseline parameter values

Description	Parameter	Orifice Pins	Porous Pin
Initial undissolved drug concentration	*b*_*0*_	775.194 kg/m^3^ (6)	505.19 kg/m^3^(5)
Rate of dissolution	*β*	10^−5^ s^−1^ (m^−3^kg)^2/3^	10^−5^ s^−1^ (m^−3^kg)^2/3^
Reaction exponent	α	2/3	2/3
Rotational velocity magnitude	*ω*	30 RPM	30 RPM
Free-diffusion coefficient	*D*_*f*_	10^−9^ m^2^/s	10^−9^ m^2^/s
Drug solubility	*S*	50 kg/m^3^	3 kg/m^3^
Permeability	*κ*	N/A	8.63 × 10^−14^ m^2^(25)
Kinematic viscosity	$v$	6.96 × 10^−7^ m^2^/s (24)	6.96 × 10^−7^ m^2^/s (24)
Orifice diameter	*d*	0.5 mm	*N/A*
Wall porosity	*ɸ*	N/A	0.17
Release medium length scale	*L*_*m*_	N/A	3 × 10^−2^ m
Effective porosity	*ɸ*_*e*_	N/A	0.9 ×*ɸ*
Effective diffusion coefficient	${D}_p={\phi}_e{D}_f/\tau$	N/A	5.1 × 10^−11^ m^2^/s
Tortuosity	$\tau$	N/A	3

The free diffusion coefficient of drug in the release medium is assumed to be of *O*(10^-9^) m^2^/s with the upper limit value of 1*×*10^-9^ m^2^/s selected for the baseline case. The effective porosity of the porous wall, ${\phi}_e$ is taken to be 90 % of the overall porosity, while the tortuosity, $\tau$, is assumed to take the value 3, which is considered an average value of the typical range of tortuosities (22). We are unaware of literature estimates of the dissolution rate, $\beta$, for these drugs. Therefore, we selected the baseline value such that the associated second Damköhler is of *O*(1) (23). The rate of stirring in the orifice pin experiments was quoted as 30 RPM, however, the rate of stirring in the porous pin experiments was not reported, therefore, we assume it to also be 30 RPM. The kinematic viscosity of the release medium (ν) was inferred from (24) while the permeability of the porous wall $\kappa$ was derived from (25).

In order to assess the effect of varying the model parameters on the resulting drug release profile, a sensitivity analysis was conducted whereby several of the model parameters were varied from the baseline values.

## Results and Discussion

### The Influence of Flow on Drug Release from the Orifice and Porous Pins

**Fig. 4:**
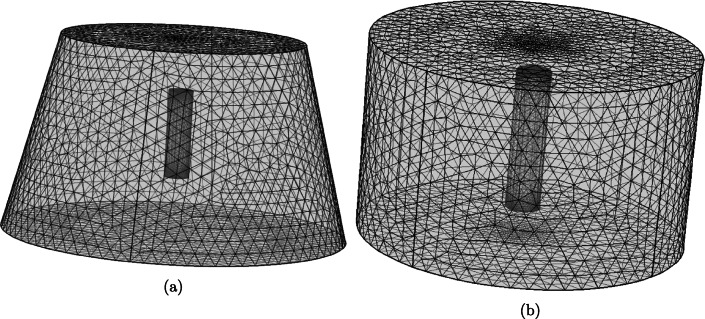
(a) FEM mesh generated for the 3D 8-orifice pin geometry for the influence of flow study. Mesh consists of 217,071 domain elements, 10,434 boundary elements, and 928 edge elements. A similar mesh for the 2-orifice pin was also created which consisted of 178,296 domain elements, 9050 boundary elements, and 728 edge elements. (b) FEM mesh generated for the 3D porous pin geometry for the influence of flow study. Mesh consists of 243,870 domain elements, 8652 boundary elements, and 652 edge elements.

Our initial study explores the extent to which the agitation of the release medium influences the release of drug from the orifice and porous pins. The baseline parameter values used in this study are shown in Table 2 and the computational meshes are shown in Fig. 4. The meshes were constructed within the software using tetrahedral elements and for both meshes, the “Normal” element size was used, calibrated for “Fluid Dynamics” physics. To aid computational accuracy in fluid/boundary interactions, three additional boundary layers were added to each surface for both meshes. Both models were solved using the iterative generalized minimal residual (GMRES) numerical method.

**Fig. 5:**
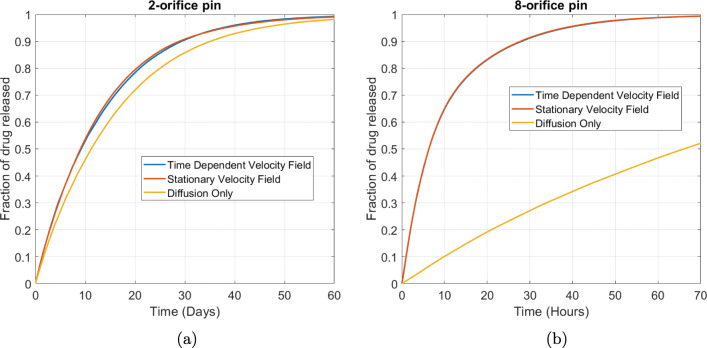
(a) Plot showing the effect of including fluid flow compared to a purely diffusive problem for (a) the 2-orifice pin and (b) the 8-orifice pin. The baseline free diffusion coefficient of ${D}_f=1{0}^{-9}\kern0.3em {\mathrm{m}}^2/\mathrm{s}$ and rotation speed of *ω* = 30 RPM have been used to generate these plots. The fraction of drug released was calculated using ${M}_{rel}(t)=\underset{\Omega_1}{\int }c\kern0.15em {d\Omega}_1/{b}_0{\int}_{\Omega_3}{d\Omega}_3$.

In Fig. 5 we display the drug release profiles resulting from the time-dependent diffusion and advection-diffusion models for the 8-orifice and 2-orifice pins. Moreover, we compare the drug release profiles when we approximate the time-dependent flow field by steady flow. Firstly, we note that there is a negligible difference between the drug release profiles assuming a time-dependent or steady velocity field, with the implication that the less-computationally intensive steady flow equations are sufficient. However, we note significant differences between the drug release profiles resulting from the diffusion and advection-diffusion models. Specifically, the inclusion of flow through the rotation of the container results in faster drug release for both the 2-orifice and the 8-orifice pin, noting the difference in scale on the time axis between the 2-orifice pin (days) and 8-orifice pin (hours) release profiles. The effect is notably larger for the 8-orifice pin, suggesting that the influence of flow becomes more prominent with increasing number of orifices.

**Fig. 6:**
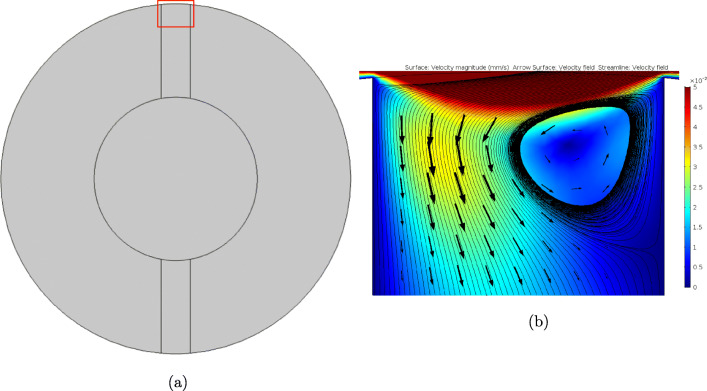
(a) A horizontal cross-sectional slice through the centre of the top pair of orifices of the 8-orifice pin. (b) Image of the velocity field within the red box region of the cross-sectional slice. The baseline rotation speed of *ω* = 30 RPM has been used to generate this plot.

To probe this further, we considered the flow field within one of the orifices of the 8-orifice pin. As can be seen in Fig. 6, the fluid flows into the orifices of the pins, reaching maximal flow speeds of *O*(10^*−*2^) mm/s, and creating interesting recirculation patterns. The typical Péclet numbers based upon the average fluid velocity magnitudes in each region of the porous pin are calculated to be:

$$ P{e}_{\Omega_1}=\frac{u_{\Omega_1}{L}_m}{D_f}\approx 2.8\times 1{0}^6,\kern1em P{e}_{\Omega_2}=\frac{u_{\Omega_2}{L}_o}{D_f}\approx 13.8-26.4,\kern1em P{e}_{\Omega_3}=\frac{u_{\Omega_3}{L}_d}{D_f}\approx \left(2.3-8340\right)\times 1{0}^{-4}, $$

where ${u}_{\Omega_1}$, ${u}_{\Omega_2}$ and ${u}_{\Omega_3}$ are the average velocity magnitudes in each domain and where a range is specified, the lower value corresponds to the 2-orifice pin while the upper value corresponds to the 8-orifice pin. The length scales utilised to compute the Péclet numbers (*L*_*m*_, *L*_*o*_ and *L*_*d*_), are chosen to be the average ‘thickness’ of the release medium (average radius of container minus radius of pin), the length of the orifices and the radius of the inner drug core, respectively. The calculations show that advection dominates over diffusion within the orifices and within the core for each orifice pin, with the effect more pronounced for the 8-orifice pin. Since we have assumed our baseline free diffusion coefficient to be the upper limit of the range of diffusion coefficients of solutes (26), our finding that the advective component of drug transport within this system is significant is likely to be valid for all physically realistic solute diffusion coefficients. In the sensitivity analysis in “Orifice Pin Sensitivity Study” section, we further explore the influence of varying the rotational velocity of the conical flask.

**Fig. 7:**
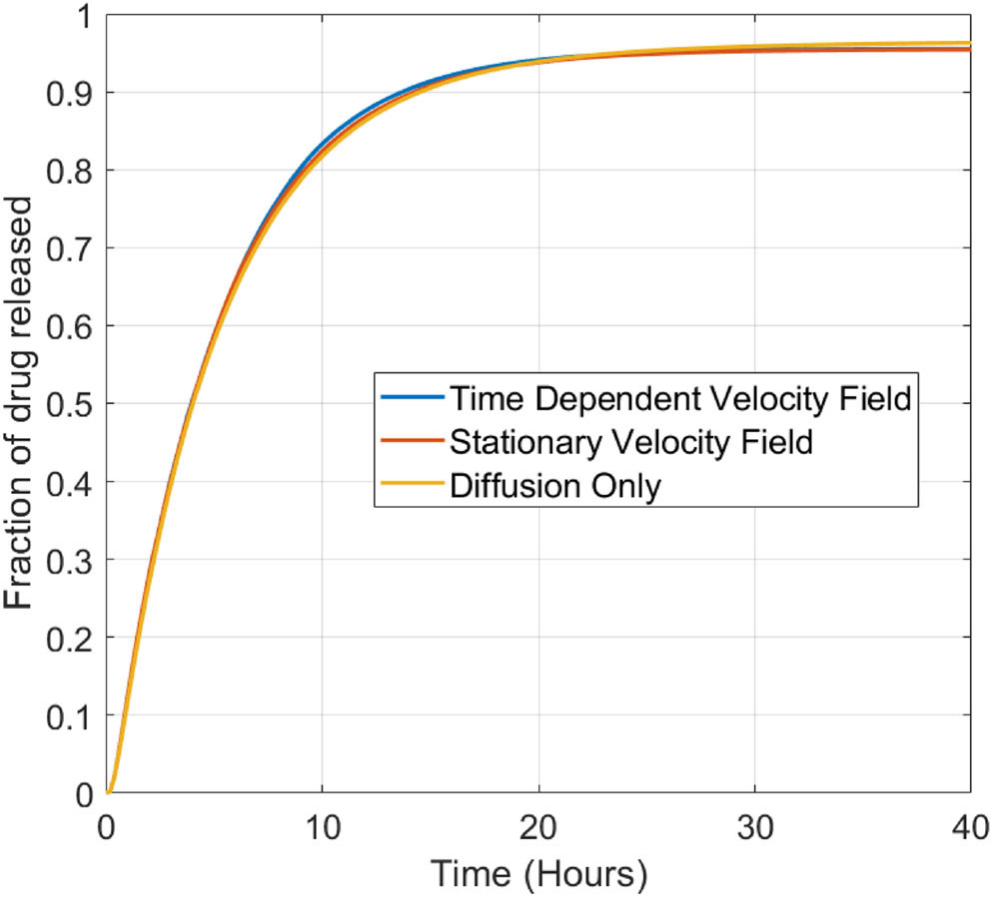
Plot showing the effect of including fluid flow compared to a purely diffusive problem for the porous pin. The baseline free diffusion coefficient of *D*_*f*_ *=* 10^−9^ m^2^/s and rotation speed of *ω* = 30 RPM have been used to generate these plots. The fraction of drug released was calculated using ${M}_{rel}(t)=\underset{\Omega_1}{\int }{c}_m{d\Omega}_1/{b}_0{\int}_{\Omega_3}{d\Omega}_3$.

In Fig. 7, we turn our attention to the influence of fluid flow on drug release from the porous pin. The results show clearly that the drug release profile is insensitive to flow, regardless of whether time-dependent or steady-flow is simulated. The implication is that the model for the porous pin can be significantly simplified by neglecting the fluid dynamics equations. We explore this further in the following Section.

### Porous Pin Model Reduction

**Fig. 8:**
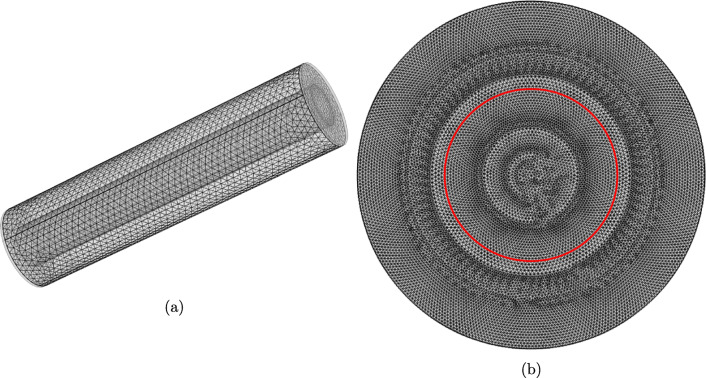
(a) FEM mesh generated for the 3D porous pin geometry. Mesh consists of 136,059 domain elements, 11,316 boundary elements, and 664 edge elements. (b) FEM mesh generated for the 2D porous pin geometry. Mesh consists of 24,930 domain elements and 472 boundary elements. The red circle indicates the interface between the inner drug core and the porous wall of the geometry.

Given that our results demonstrate that fluid flow may reasonably be neglected for the porous pin, we now explore whether the cylindrical symmetry of the porous pin and release medium (Fig. 3a) lends itself to further model reduction, with potential benefits including a reduction in computational cost and the possibility of obtaining analytical solutions. Specifically, in addition to the 3D diffusion model, we consider the corresponding 2D and 1D diffusion models. For simplicity we approximate the release medium by an infinite sink boundary condition. Each of the models in this study were solved using the GMRES iterative numerical method. It should be noted that the 2D and 3D models were solved in the software’s default Cartesian coordinate system, whilst the 1D model was solved in a cylindrical coordinate system, with diffusion modelled only in the radial direction. For both the 3D and 2D models, the generated meshes were constructed using the “Extremely Fine” mesh setting in COMSOL Multiphysics®, version 5.3a. This produced the meshes shown in Fig. 8, using tetrahedral elements for the 3D mesh and triangular elements for the 2D mesh. The 1D model, consisting purely of an interval, was also discretised using the “Extremely Fine” mesh setting, resulting in an interval of 101 equally spaced points.

**Fig. 9:**
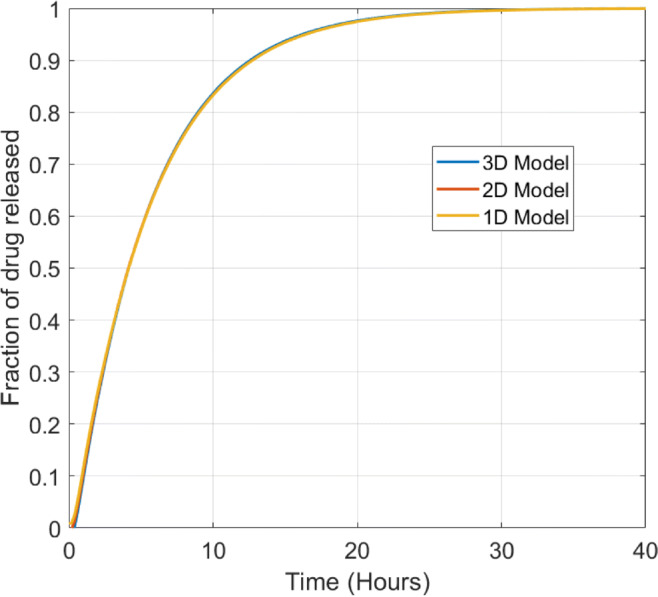
Plot showing fraction of drug released for the porous pin for the 1D, 2D and 3D diffusion models using the relevant baseline parameters from Table 2. The fraction of drug released in the case of the 3D model was calculated using ${M}_{rel}(t)=1-\left(\underset{\Omega_3}{\int }{c}_d{d\Omega}_3+\underset{\Omega_2}{\int }{c}_p{d\Omega}_2\right)/{b}_0{\int}_{\Omega_3}{d\Omega}_3$.

In Fig. 9 we observe that the release profiles generated from the 1D, 2D and 3D models are barely distinguishable, confirming that drug release from the pin may be simplified as a 1D problem.

### Sensitivity Analysis

In this Section we conduct a sensitivity analysis to assess the effect of varying the key model parameters on the resulting drug release profile for both the orifice pins and the porous pin. Dissolution is now included in the models so that for the orifice pins we simulate the 3D dissolution-diffusion-advection model with steady flow, while for the porous pin we simulate the 1D dissolution-diffusion model. The baseline parameter values used in this study are shown in Table 2, while the range of parameter values considered are provided in the Supplementary Data. In addition to the Péclet numbers reported in “The Influence of Flow on Drug Release from the Orifice and Porous Pins” section, in order to help rationalise the results, we also calculate the first and second Damköhler numbers (*Da*_*I*_ and *Da*_*II*_ ) as well as the normalised solubility *S*^*∗*^ for the orifice and porous pins. These numbers are defined as follows:

$$ D{a}_I=\frac{L_d\beta {b}_0^{2/3}}{u_{\Omega_3}},\kern1em D{a}_{II}=\frac{L_d^2\beta {b}_0^{2/3}}{D_f},\kern1em {S}^{\ast }=\frac{S}{b_0}. $$

These numbers are tabulated along with the times for 50% and 95% drug release in the Supplementary Data.

#### Numerical Solution

The 2 and 8-orifice pin meshes used in this study are similar to those shown in Fig. 4a, including the use of tetrahedral elements. However, the resultant meshes were chosen to be considerably denser in this study due to (i) the wide variation in parameter values to be considered; (ii) the inclusion of the drug dissolution process and; (iii) the multiple timescales for drug release that will be encountered. In the case of the 2-orifice pin, the final mesh consisted of 969,889 domain elements, 18,094 boundary elements, and 1052 edge elements and the 8-orifice pin mesh consisted of 1,094,980 domain elements, 21,520 boundary elements, and 1404 edge elements. These meshes were created using the “Finer” mesh setting, calibrated for “Fluid Dynamics” problems. Both the 2 and 8-orifice pin models were solved using the GMRES method. In the case of the porous pin a 1D interval was created and discretised into 2500 equally spaced points. Lastly, the 1D porous pin model was solved using the PARDISO direct numerical method.

#### Orifice Pin Sensitivity Study

**Fig. 10:**
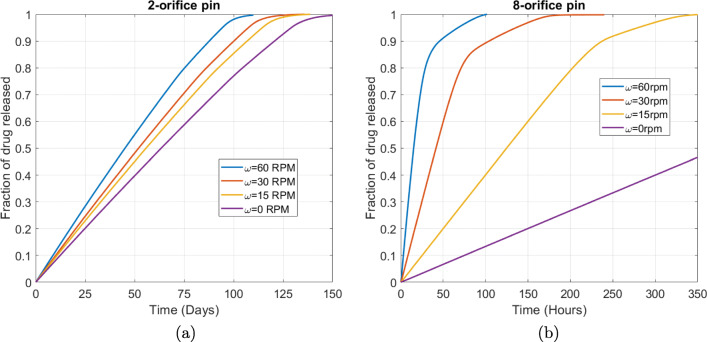
Plots showing the effect of varying the rotational flow speed for (a) the 2-orifice pin and (b) the 8-orifice pin. The remaining parameters are the baseline parameters as stated in Table 2. The fraction of drug released was calculated using. ${M}_{rel}(t)=\underset{\Omega_1}{\int }c\kern0.15em {d\Omega}_1/{b}_0\underset{\Omega_3}{\int }{d\Omega}_3$.

We start by varying the rotation speed from 0-60 RPM (Fig. 10). As expected, the faster the rotation, the quicker the drug release. However, the effect is more pronounced for the 8-orifice pin compared with the 2-orifice pin: doubling the rotation speed results in the time for 95% drug released to be approximately halved for the 8-orifice pin, yet only reduced by a factor of approximately 1.1 for the 2-orifice pin. Note again the difference in scale on the time axis between the 2-orifice pin (days) and 8-orifice pin (hours) release profiles, which will be a recurrent feature in this sensitivity analysis. While $P{e}_{\Omega_3}$ increases with rotation speed for each pin, diffusion remains the fastest transport process for the 2-orifice pin, with $P{e}_{\Omega_3}$ only increasing above 1 for the 8-orifice pin for the fastest rotation speed considered. Taken together, these results demonstrate that rotation speed should be an important consideration and chosen carefully to take account of the pin geometry (in this case the number of orifices).

**Fig. 11:**
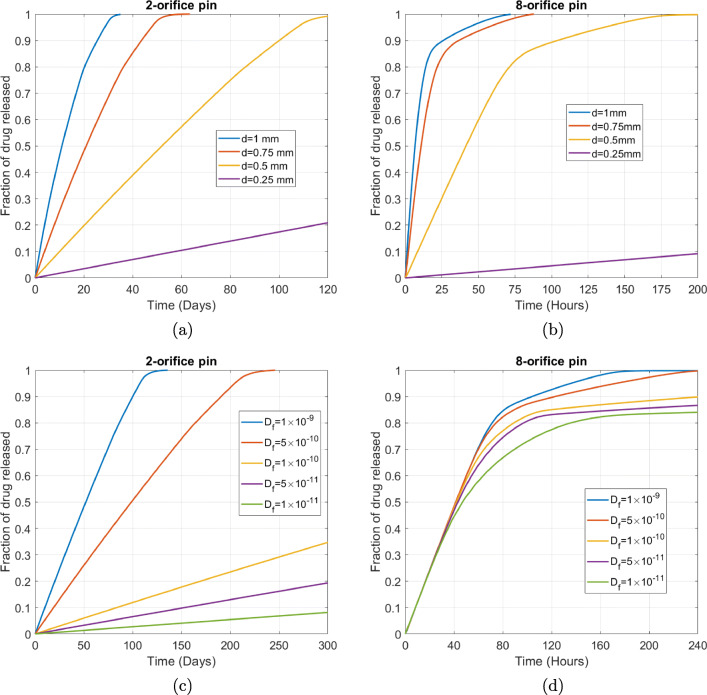
Plots showing the effect of varying the orifice diameter *d* for (a) the 2-orifice pin and (b) the 8-orifice pin. Plots showing the effect of varying the drug free diffusion coefficient *D*_*f*_ for (c) the 2-orifice pin and (d) the 8-orifice pin. The remaining parameters are baseline parameters as stated in Table 2. The fraction of drug released was calculated using ${M}_{rel}(t)=\underset{\Omega_1}{\int }c\kern0.15em {d\Omega}_1/{b}_0\underset{\Omega_3}{\int }{d\Omega}_3$

In Fig. 11a and b we vary the diameter of the orifices *d*. As one might expect, drug is released more quickly with increasing diameter of the orifices. It is notable for the 8-orifice pin that the difference between release profiles reduces markedly with increasing orifice diameter. In particular, a relatively small difference in release profile is observed between the 1 mm and 0*.*75 mm diameter orifices. Analysis of the non-dimensional numbers reveals these larger orifice diameters result in *Da*_*I*_ *<* 1 , indicating that the time scale associated with advection is smaller than reaction: in other words, the dissolution of drug is becoming the rate limiting step and increasing the orifice diameters further will have an increasingly negligible effect on the release profile.

In Fig. 11c and d we vary the free drug diffusion coefficient *D*_*f*_. Ultimately, for each of the 2-orifice and 8-orifice pins, decreasing *D*_*f*_ results in slower drug release. The effect of reducing *D*_*f*_ is more pronounced for the 2-orifice pin, where diffusion remains the fastest transport process as evidenced by $P{e}_{\Omega_3}$*<<* 1 for all values of ${D}_f$ considered. However, for the 8-orifice pin, reducing ${D}_f$ has a much smaller influence on the release profile since decreasing ${D}_f$ consistently results in $P{e}_{\Omega_3}$*>* 1, meaning that advection is the fastest transport process. Interestingly, for the 8-orifice pin, the release profiles are remarkably similar until around 40% of the drug is released. A probable explanation is that the medium is fully saturated during this initial period and since advection is the fastest transport process, reducing ${D}_f$ has a negligible effect on the rate at which the dissolved drug is released. The point at which the release profiles diverge likely corresponds to the time taken for the dissolved drug concentration to fall back below the saturation level.

**Fig. 12:**
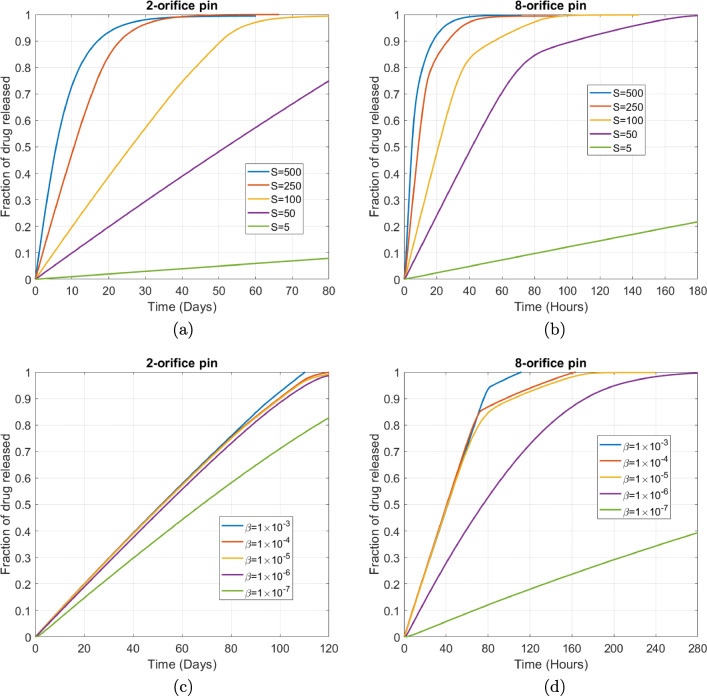
Plots showing the effect of varying the drug solubility *S* for (a) the 2-orifice pin and (b) the 8-orifice pin. Plots showing the effect of varying the dissolution rate *β* for (c) the 2-orifice pin and (d) the 8-orifice pin. The remaining parameters are the baseline parameters as stated in Table 2. The fraction of drug released was calculated using ${M}_{rel}(t)=\underset{\Omega_1}{\int }c\kern0.15em {d\Omega}_1/{b}_0\underset{\Omega_3}{\int }{d\Omega}_3$.

In Fig. 12a and b we vary the drug solubility *S*. In essence, with increasing solubility, more drug is able to dissolve more quickly. It is evident that drug release from each pin is highly sensitive to changes in *S*: when the drug solubility is increased 10-fold, the 95% drug release time reduces approximately 5-fold for the 2-orifice and 8-orifice pins. Conversely, when the drug solubility is decreased 10-fold, the 95% drug release time increases approximately 10-fold. Since S^*^< 1 in all cases considered (i.e. the solubility is less than the initial drug loading concentration) the solubility is release-rate limiting until the undissolved drug concentration falls below the solubility of drug in the release medium, after which the transport processes of diffusion and advection govern the release rate, provided the dissolution rate is sufficiently fast. This likely explains the transition from linear to nonlinear release rate as shown in Fig. 12a and b.

In Fig. 12c and d we vary the drug dissolution rate *β*. It is notable that as we increase *β* the effect on the drug release profile becomes increasingly negligible, particularly so for the 2-orifice pin. The rationale for this is that while increasing *β* results in both *Da*_*I*_ and *Da*_*II*_ becoming increasingly greater than 1, indicating that the dissolution of drug becomes the fastest process, S^*^ remains much less than 1 for all cases. In other words, the drug release becomes limited by the solubility of the drug in the release medium, rather than the rate of dissolution. For sufficiently high *β*, the medium becomes saturated and more drug is only able to dissolve following the time scale over which the transport processes (diffusion and advection) are able to release drug from the system. For high *β*, where the curves are barely distinguishable for a large portion of the release, the point of divergence likely relates to the point where drug solubility is no longer rate-controlling.

#### Porous Pin Sensitivity Analysis

**Fig. 13:**
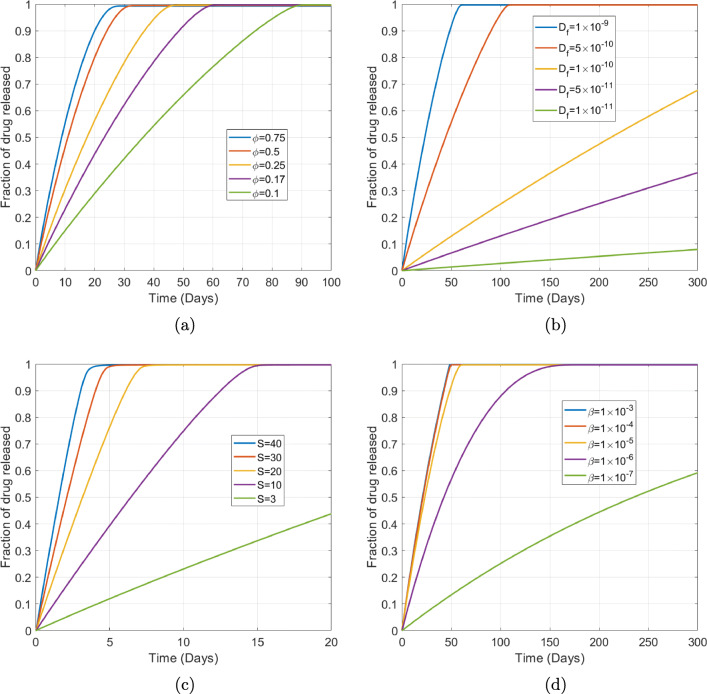
Plots showing the effect of varying the following porous pin model parameters (a) porosity of the porous wall *ɸ*, (b) free drug diffusion coefficient ${D}_f$, (c) drug solubility *S* and (d) drug dissolution rate *β*. The remaining parameters are baseline parameters as stated in Table 2. The fraction of drug released in all cases was calculated using ${M}_{rel}(t)={\int}_{L_d+{L}_p}^{L_d+{L}_p+{L}_m}{c}_m rdr/{b}_0{\int}_0^{L_d} rdr$.

While the general trends observed when varying parameters in the orifice pin model are qualitatively the same for the corresponding parameters in the porous pin model, a separate sensitivity analysis is warranted in the case of the porous pin since flow is neglected. In Fig. 13a we observe that increasing the porosity of the porous wall results in faster drug release. While the material properties, manufacturing techniques and application area will ultimately dictate the range of porosities possible, varying the porosity over the range 0*.*1 *−* 0*.*75 results in 95% release times of 81 *−* 23 days, demonstrating that the results are relatively sensitive to this parameter. The reduction in drug released with decreasing diffusion coefficient shown in Fig. 13b is a direct result of the increase in the timescale for diffusion, which governs the rate at which dissolved drug is transported out of the pin. In Fig. 13c we examine the effect on drug release of varying drug solubility *S* and observe that a ten-fold increase in solubility results in an approximately 4-fold decrease in 95% drug release time. For all values of *S* considered, S^*^<< 1 indicating that solubility is the rate-limiting step. As *S* is increased, more drug dissolves more quickly, after which point drug transport out of the system is governed by diffusion. In Figure Fig. 13d we observe that increasing the dissolution rate *β* results in faster drug release. Similarly to the orifice pins, we observe that there becomes a point where increasing *β* further has a negligible impact on the drug release. In this case the drug is able to dissolve more quickly than it is able to diffuse out of the system, such that a combination of the drug solubility and diffusion govern the rate the of release.

**Fig. 14:**
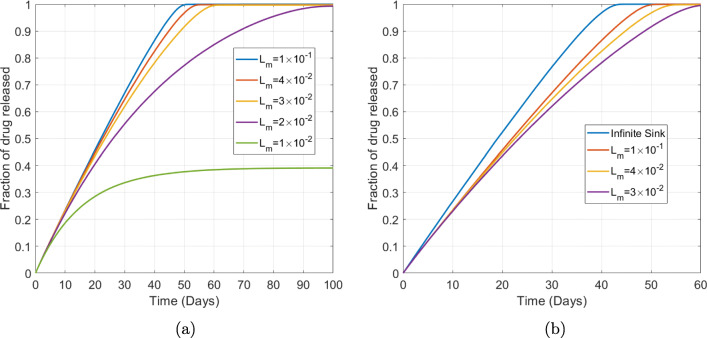
Plots showing the effect of (a) varying the length scale of the release medium (b) approximating the release medium through an infinite sink boundary condition. The remaining parameters are baseline parameters as stated in Table 2. The fraction of drug released in all cases was calculated using ${M}_{rel}(t)={\int}_{\left({L}_d+{L}_p\right)}^{\left({L}_d+{L}_p+{L}_m\right)}{c}_m rdr/{b}_0{\int}_0^{L_d} rdr$, except for the infinite sink case where ${M}_{rel}(t)=1-\left({\int}_0^{L_d}{c}_d rdr+\phi {\int}_{L_d}^{\left({L}_d+{L}_p\right)}{c}_p rdr\right)/{b}_0\phi {\int}_0^{L_d} rdr$ was used.

Given that we have already demonstrated the ability to reduce the porous pin model from 3D to 1D, it is also of interest to explore the effect of varying the length scale *L*_*m*_ associated with the release medium (related the volume of the release medium and ultimately the dimensions of the release container) with a view to approximating the release medium as an infinite sink boundary condition. In Fig. 14a we demonstrate that selecting *L*_*m*_ to be sufficiently small results in incomplete drug release as a result of the drug becoming saturated in the release medium: further drug release would only be possible if the release medium was replenished. On the other hand, as we increase *L*_*m*_ the release profiles become increasingly similar, suggesting that the drug concentration in the release medium is becoming sufficiently small such that the concentration gradient between drug inside the porous wall and drug in the release medium is maintained at a similar level. In Fig. 14b we consider the extreme case of an infinite sink boundary condition and show that simplifying the model in this way may overestimate the drug release.

### Limitations

The models we have described make a number of assumptions as described in the preceding text. The most notable assumptions relate to the dissolution process. We assume that fluid is able to infiltrate the cores of the pins rapidly such that the drug becomes instantly fully wetted, meaning that the dissolution process is initiated throughout the entire core. In reality, flow will likely take a finite time to penetrate into the core and a more accurate description of the dissolution process may involve a moving dissolution front. This assumption also has implications in terms of the description of fluid flow that we employ within the drug core. Specifically, the Navier-Stokes equations may not represent the most appropriate description of the fluid dynamics while the drug is dissolving. Taken together, these model assumptions may lead to overestimation of the drug release profile at sufficiently early times during which the drug is still dissolving, but should be valid in the case of rapid dissolution.

We acknowledge that the fluid environment created in *in vitro* experiments may well be different from those found in *in vivo* experiments. While it is desirable to test DFIs in the most realistic environment possible, simple *in vitro* testing, as described in this paper, is still ubiquitous in the literature. This does not affect the findings and conclusions of our work, which highlight the importance of considering the fluid environment when assessing drug release from DFIs.

## Conclusions

In this paper, we have developed a series of multiphysics mathematical models to explore the influence of fluid flow on drug release from two seemingly similar DFIs: a porous pin and an orifice pin. We have established that the importance of fluid flow on the drug release profile varies substantially between the two pins, owing to their differing porous properties. In particular, a porous wall pin with pores on the order of *μ*m is insensitive to the fluid flow environment in the release medium, whereas a pin with orifices of the order of mm leads to not insignificant fluid flow within the device and ultimately has a substantial influence on the drug release profile. We have demonstrated that the porous pin model may be simplified to a radial 1D dissolution-diffusion model while a 3D dissolution-advection-diffusion model is required to accurately describe the drug release from the orifice pins, albeit with the approximation of steady flow being reasonable. Through a thorough sensitivity analysis, we have shown that the balance of reaction-advection-diffusion in terms of key nondimensional numbers is critical in determining the overall release rate of the drug. Our findings potentially have important implications in terms of devising the optimal experimental protocol for quantifying drug release from DFIs.

### Acknowledgments and Disclosures

David King would like to gratefully acknowledge the funding provided by EPSRC (grant number EP/M506539/1). Sean McGinty acknowledges funding provided by EPSRC (grant number EP/S030875/1). The authors declare that they have no conflicts of interest.

## Supplementary information

ESM 1 (DOCX 87.3 kb)
