# Supplementary material for: How Does Fluid Flow Influence Drug Release from Drug Filled Implants?
Source: Pharm Res. 2022 Jan 7;39(1):25–40. doi: 10.1007/s11095-021-03127-4 (PMC8837542; doi:10.1007/s11095-021-03127-4)
Supplement: Supplementary file 1 — (DOCX 87.3 kb) [file 11095_2021_3127_MOESM1_ESM.docx]

**A Supplementary data**

**A.1 Porous pin sensitivity analysis**

|  | **Second Damk¨ohler number *DaI I*** | **Normalised solubility**  ***S^*^*** | **Time for 50 %**  **release (Days)** | **Time for 95 %**  **release (Days)** |
| --- | --- | --- | --- | --- |
| **Baseline** | **1.6** | **0.0059** | **23** | **53** |
| Parameter | Second Damk¨ohler number *DaI I* | Normalised solubility  *S∗* | Time for 50 %  release (Days) | Time for 95 %  release (Days) |
| *β* = 10*−*7 | 0*.*016 | 0*.*0059 | 235 | 822 |
| *β* = 10*−*6 | 0*.*16 | 0*.*0059 | 43 | 122 |
| *β* = 10*−*4 | 16 | 0*.*0059 | 21 | 46 |
| *β* = 10*−*3 | 157 | 0*.*0059 | 21 | 45 |
| *Df* = 10*−*11 | 157 | 0*.*0059 | 2088 | 4532 |
| *Df* = 5 *×* 10*−*11 | 31 | 0*.*0059 | 421 | 911 |
| *Df* = 10*−*10 | 16 | 0*.*0059 | 212 | 460 |
| *Df* = 5 *×* 10*−*10 | 3*.*1 | 0*.*0059 | 44 | 98 |
| *Lm* = 10*−*2 | 1*.*6 | 0*.*0059 | N/A | N/A |
| *Lm* = 2 *×* 10*−*2 | 1*.*6 | 0*.*0059 | 26 | 79 |
| *Lm* = 4 *×* 10*−*2 | 1*.*6 | 0*.*0059 | 23 | 48 |
| *Lm* = 10*−*1 | 1*.*6 | 0*.*0059 | 22 | 45 |
| *ϕ* = 0*.*1 | 1*.*6 | 0*.*0059 | 36 | 81 |
| *ϕ* = 0*.*25 | 1*.*6 | 0*.*0059 | 17 | 41 |
| *ϕ =* 0*.*5 | 1*.*6 | 0*.*0059 | 11 | 27 |
| *ϕ* = 0*.*75 | 1*.*6 | 0*.*0059 | 9 | 23 |
| *S* = 10 | 1*.*6 | 0*.*019 | 6 | 14 |
| *S* = 20 | 1*.*6 | 0*.*039 | 3 | 7 |
| *S* = 30 | 1*.*6 | 0.059 | 2 | 4 |
| *S* = 40 | 1*.*6 | 0*.*079 | 2 | 3 |

Table 3: Porous pin sensitivity analysis. All values are approximate. Parameter units are shown in Table 2.

**A.2 2-orifice pin sensitivity analysis**

| **2-Orifice Pin** | **Time for 50 %**  **release (Days)** | **Time for 95 %**  **release (Days)** |
| --- | --- | --- |
| **Baseline** | **52** | **107** |
| Parameter | Time for 50 %  release (Days) | Time for 95 %  release (Days) |
| *Df* = 10*−*11 | 1958 | 4241 |
| *Df* = 5 *×* 10*−*11 | 810 | 1707 |
| *Df* = 10*−*10 | 441 | 923 |
| *Df* = 5 *×* 10*−*10 | 99 | 205 |
| *β* = 10*−*7 | 68 | 147 |
| *β* = 10*−*6 | 54 | 111 |
| *β* = 10*−*4 | 52 | 107 |
| *β* = 10*−*3 | 52 | 103 |
| *d* = 0*.*25 *×* 10*−*3 | 289 | 560 |
| *d* = 0*.*75 *×* 10*−*3 | 21 | 48 |
| *d* = 1 *×* 10*−*3 | 11 | 29 |
| *ω* = 15 | 56 | 114 |
| *ω* = 60 | 45 | 94 |
| *S* = 5 | 537 | 1140 |
| *S* = 100 | 26 | 56 |
| *S* = 250 | 11 | 28 |
| *S* = 500 | 6 | 22 |

Table 4: 2-orifice pin sensitivity analysis displaying times for 50% and 95% release. All values are approximate. Parameter units are shown in Table 2.

*S*

Table 5: 2-orifice pin sensitivity analysis displaying nondimensional numbers. All values are approximate. Parameter units are shown in Table 2.

| **2-Orifice Pin** | **First Damk¨ohler number *DaI*** | **Second Damk¨ohler number *DaI I*** | **Normalised solubility *S∗*** | **P´eclet number *P e***  **Ω1** | **P´eclet number *P e***  **Ω2** | **P´eclet number *P e***  **Ω3** |
| --- | --- | --- | --- | --- | --- | --- |
| **Baseline** | **7325***.***9** | **1***.***654** | **0***.***0645** | **2***.***81** *×* **106** | **13***.***75** | **2***.***26** *×* **10***−***4** |
| Parameter | First Damk¨ohler number *DaI* | Second Damk¨ohler number *DaI I* | Normalised solubility  *∗* | P´eclet number *P e*  Ω1 | P´eclet number *P e*  Ω2 | P´eclet number *P e*  Ω3 |
| *Df* = 10*−*11 | 7445 | 165*.*4 | 0*.*0645 | 2*.*81 *×* 108 | 1375 | 2*.*22 *×* 10*−*2 |
| *Df* = 5 *×* 10*−*11 | 7086 | 33*.*1 | 0*.*0645 | 5*.*62 *×* 107 | 275 | 4*.*67 *×* 10*−*3 |
| *Df* = 10*−*10 | 7100 | 16*.*54 | 0*.*0645 | 2*.*81 *×* 107 | 137*.*5 | 2*.*33 *×* 10*−*3 |
| *Df* = 5 *×* 10*−*10 | 7106 | 3*.*31 | 0*.*0645 | 5*.*62 *×* 106 | 27*.*5 | 4*.*66 *×* 10*−*4 |
| *β* = 10*−*7 | 73*.*3 | 0*.*01654 | 0*.*0645 | 2*.*81 *×* 106 | 13*.*75 | 2*.*26 *×* 10*−*4 |
| *β* = 10*−*6 | 732*.*6 | 0*.*1654 | 0*.*0645 | 2*.*81 *×* 106 | 13*.*75 | 2*.*26 *×* 10*−*4 |
| *β* = 10*−*4 | 73259 | 16*.*54 | 0*.*0645 | 2*.*81 *×* 106 | 13*.*75 | 2*.*26 *×* 10*−*4 |
| *β* = 10*−*3 | 732590 | 165*.*4 | 0*.*0645 | 2*.*81 *×* 106 | 13*.*75 | 2*.*26 *×* 10*−*4 |
| *d* = 0*.*25 *×* 10*−*3 | 34478 | 1*.*654 | 0*.*0645 | 2*.*81 *×* 106 | 0*.*138 | 4*.*8 *×* 10*−*5 |
| *d* = 0*.*75 *×* 10*−*3 | 11717 | 1*.*654 | 0*.*0645 | 2*.*81 *×* 106 | 35*.*4 | 1*.*41 *×* 10*−*4 |
| *d* = 1 *×* 10*−*3 | 1029 | 1*.*654 | 0*.*0645 | 2*.*81 *×* 106 | 65*.*1 | 1*.*61 *×* 10*−*3 |
| *ω* = 15 | 87235 | 1*.*654 | 0*.*0645 | 1*.*4 *×* 106 | 6*.*36 | 1*.*9 *×* 10*−*5 |
| *ω* = 60 | 348 | 1*.*654 | 0*.*0645 | 5*.*62 *×* 106 | 31*.*2 | 4*.*75 *×* 10*−*3 |
| *S* = 5 | 7326 | 1*.*654 | 0*.*00645 | 2*.*81 *×* 106 | 13*.*75 | 2*.*26 *×* 10*−*4 |
| *S* = 100 | 7326 | 1*.*654 | 0*.*129 | 2*.*81 *×* 106 | 13*.*75 | 2*.*26 *×* 10*−*4 |
| *S* = 250 | 7326 | 1*.*654 | 0*.*323 | 2*.*81 *×* 106 | 13*.*75 | 2*.*26 *×* 10*−*4 |
| *S* = 500 | 7326 | 1*.*654 | 0*.*645 | 2*.*81 *×* 106 | 13*.*75 | 2*.*26 *×* 10*−*4 |

**A.3 8-orifice pin sensitivity analysis**

| **8-Orifice Pin** | **Time for 50 %**  **release (Hours)** | **Time for 95 %**  **release (Hours)** |
| --- | --- | --- |
| **Baseline** | **42** | **136** |
| Parameter | Time for 50 %  release (Hours) | Time for 95 %  release (Hours) |
| *Df* = 10*−*11 | 47 | 2239 |
| *Df* = 5 *×* 10*−*11 | 43 | 725 |
| *Df* = 10*−*10 | 42 | 418 |
| *Df* = 5 *×* 10*−*10 | 41 | 173 |
| *β* = 10*−*7 | 372 | 1083 |
| *β* = 10*−*6 | 75 | 202 |
| *β* = 10*−*4 | 41 | 127 |
| *β* = 10*−*3 | 42 | 85 |
| *d* = 0*.*25 *×* 10*−*3 | 1092 | 2105 |
| *d* = 0*.*75 *×* 10*−*3 | 11 | 56 |
| *d* = 1 *×* 10*−*3 | 7 | 43 |
| *ω* = 15 | 125 | 279 |
| *ω* = 60 | 15 | 67 |
| *S* = 5 | 426 | 1398 |
| *S* = 100 | 21 | 71 |
| *S* = 250 | 9 | 35 |
| *S* = 500 | 5 | 24 |

Table 6: Release times for 50 % and 95 % across a variety of parameter ranges for the 8-orifice pin. All values are approximate and rounded. Parameter units are shown in Table 2.

| **8-Orifice Pin** | **First Damk¨ohler number *DaI*** | **Second Damk¨ohler number *DaI I*** | **Normalised solubility *S∗*** | **P´eclet number *P e***  **Ω1** | **P´eclet number *P e***  **Ω2** | **P´eclet number *P e***  **Ω3** |
| --- | --- | --- | --- | --- | --- | --- |
| **Baseline** | **1***.***98** | **1***.***654** | **0***.***0645** | **2***.***81** *×* **106** | **26***.***37** | **0***.***834** |
| Parameter | First Damk¨ohler number *DaI* | Second Damk¨ohler number *DaI I* | Normalised solubility *S∗* | P´eclet number *P e*  Ω1 | P´eclet number *P e*  Ω2 | P´eclet number *P e*  Ω3 |
| *Df* = 10*−*11 | 1*.*98 | 165*.*4 | 0*.*0645 | 2*.*81 *×* 108 | 2636*.*6 | 83*.*4 |
| *Df* = 5 *×* 10*−*11 | 1*.*98 | 33*.*1 | 0*.*0645 | 5*.*62 *×* 107 | 527*.*3 | 16*.*7 |
| *Df* = 10*−*10 | 1*.*98 | 16*.*54 | 0*.*0645 | 2*.*81 *×* 107 | 263*.*66 | 8*.*34 |
| *Df* = 5 *×* 10*−*10 | 1*.*98 | 3*.*31 | 0*.*0645 | 5*.*62 *×* 106 | 52*.*73 | 1*.*67 |
| *β* = 10*−*7 | 0*.*0198 | 0*.*0165 | 0*.*0645 | 2*.*81 *×* 106 | 26*.*37 | 0*.*834 |
| *β* = 10*−*6 | 0*.*198 | 0*.*165 | 0*.*0645 | 2*.*81 *×* 106 | 26*.*37 | 0*.*834 |
| *β* = 10*−*4 | 19*.*8 | 16*.*54 | 0*.*0645 | 2*.*81 *×* 106 | 26*.*37 | 0*.*834 |
| *β* = 10*−*3 | 198*.*3 | 165*.*4 | 0*.*0645 | 2*.*81 *×* 106 | 26*.*365 | 0*.*834 |
| *d* = 0*.*25 *×* 10*−*3 | 62*.*4 | 1*.*654 | 0*.*0645 | 2*.*81 *×* 106 | 2*.*52 | 0*.*0265 |
| *d* = 0*.*75 *×* 10*−*3 | 0*.*367 | 1*.*654 | 0*.*0645 | 2*.*81 *×* 106 | 62*.*04 | 4*.*5 |
| *d* = 1 *×* 10*−*3 | 0*.*13 | 1*.*654 | 0*.*0645 | 2*.*81 *×* 106 | 104*.*2 | 12*.*74 |
| *ω* = 15 | 6*.*39 | 1*.*654 | 0*.*0645 | 1*.*40 *×* 106 | 10*.*25 | 0*.*26 |
| *ω* = 60 | 0*.*611 | 1*.*654 | 0*.*0645 | 5*.*62 *×* 106 | 72*.*13 | 2*.*71 |
| *S* = 5 | 1*.*98 | 1*.*654 | 0*.*00645 | 2*.*81 *×* 106 | 26*.*37 | 0*.*834 |
| *S* = 100 | 1*.*98 | 1*.*654 | 0*.*129 | 2*.*81 *×* 106 | 26*.*37 | 0*.*834 |
| *S* = 250 | 1*.*98 | 1*.*654 | 0*.*3225 | 2*.*81 *×* 106 | 26*.*37 | 0*.*834 |
| *S* = 500 | 1*.*98 | 1*.*654 | 0*.*645 | 2*.*81 *×* 106 | 26*.*37 | 0*.*834 |

Table 7: 8-orifice pin sensitivity analysis displaying nondimensional numbers. All values are approximate. Parameter units are shown in Table 2.
